# CCL2: A double-edged sword in neuroblastoma, with a critical role in MYCN-amplified tumors

**DOI:** 10.1016/j.tranon.2026.102746

**Published:** 2026-04-04

**Authors:** Léo Jannot, Alexia Gazeu, Nathalie Bendriss-Vermare, Cecile Pochon, Hervé Sartelet

**Affiliations:** a INSERM, U1256, NGERE - University of Lorraine, Vandoeuvre-lès-Nancy, France; b Université de Lorraine, Nancy, France; c Department of pathology, Institut de Biopathologie Est, HCL, Université de Lyon, France; d Université de Lyon, Université Claude Bernard Lyon 1, INSERM 1052, CNRS 5286, Centre Léon Bérard, Cancer Research Center of Lyon, Lyon, 69008, France; e Department of pediatric oncology, CHRU Nancy Brabois, Université de Nancy, France; f Department of pathology, CHRU Nancy Brabois, Université de Nancy, France

**Keywords:** Neuroblastoma, Dendritic cells, Microenvironment, MYCN amplification, Opsoclonus myoclonus syndrome, CCL2

## Abstract

Neuroblastoma (NB) is the most common extracranial solid tumor in children, characterized by significant clinical heterogeneity and immune evasion. *MYCN* oncogene amplification is a major driver of tumor aggressiveness and poor prognosis and is inversely correlated with immune infiltration in the tumor microenvironment (TME). Emerging evidence highlights the pivotal role of the chemokine C-C motif ligand 2 (CCL2) in modulating the immune landscape of NB. CCL2 influences the recruitment of various immune cells, including invariant natural killer T (iNKT) cells, dendritic cells (DCs), monocytes, macrophages, and regulatory T cells (Tregs), thereby shaping either pro- or anti-tumor responses depending on the context.

*MYCN*-amplified tumors display reduced CCL2 expression, resulting in limited immune cell recruitment and the establishment of a “cold” TME with poor immune surveillance. In contrast, non-amplified tumors exhibit higher levels of CCL2, which attracts both anti-tumor immune cells such as DCs and macrophages, as well as pro-tumor populations including Tregs and tumor-associated macrophages (TAMs).

These observations underscore the dual and context-dependent role of CCL2 in NB pathogenesis. Therapeutically, targeting the CCL2/CCR2 axis has shown promise in preclinical models, including approaches to enhance CAR-T cell trafficking and reduce TAM-mediated immunosuppression.

Overall, CCL2 emerges as a central immunomodulatory molecule in NB, tightly linked to *MYCN* status and the composition of the TME. Understanding its complex biology is critical for the development of novel immunotherapies aimed at restoring effective anti-tumor immune responses, particularly in high-risk *MYCN*-amplified NB. Targeting the CCL2 axis represents a promising strategy to improve NB patient outcomes.

## Highlights

• In neuroblastoma, MYCN amplification suppresses CCL2, creating an immune-cold microenvironment.
• CCL2 shapes immune cell recruitment and balance in neuroblastoma tumors.
• Low CCL2 limits immune surveillance in high-risk MYCN-amplified NB.
• CCL2/CCR2 targeting improves CAR-T trafficking and reduces TAM suppression.

## Introduction

Neuroblastoma (NB) is the most common extracranial solid tumor in children, accounting for approximately 15% of pediatric oncology-related deaths. It arises from neural crest cells and typically develops in the adrenal glands or along the sympathetic chain. The estimated prevalence is approximately 1 in 7,000 live births. The median age at diagnosis is 19 months, with 90% of cases diagnosed before the age of five [1]. It accounts for approximately 15% of all pediatric oncology-related deaths. It is characterized by a highly heterogeneous clinical course, ranging from spontaneously regressing low-risk tumors to high-risk forms associated with poor prognosis and an overall survival rate below 50% [1]. Prognosis is influenced by multiple factors, including age at diagnosis, tumor location, *MYCN* oncogene amplification, and the feasibility of complete surgical resection in localized cases. Additional prognostic indicators include metastatic status, histopathological features, and distinct biological characteristics.

High-risk NB often exhibit specific genetic abnormalities, deletion at chromosome arms 1p, 3p, 4p and 11q, and gain of 1q, 2p or 17q, or the presence of two or more of the following: 1q, 17p, 19q, ATRX deletion and/or telomerase reverse transcriptase aberrations [2], but the most important is amplification of the *MYCN* oncogene, which are key indicators of tumor aggressiveness [3]. This *MYCN* amplification is one of the most critical molecular markers associated with poor prognosis in NB.

Located on chromosome 2p24, *MYCN* encodes a transcription factor that belongs to the MYC family, which regulates cell proliferation, differentiation, and apoptosis [2]. *MYCN* amplification occurs in approximately 20–25% of primary NB and is strongly correlated with advanced disease stage, rapid tumor progression, and poor clinical outcome. It serves as both a diagnostic and prognostic biomarker and plays a pivotal role in the oncogenesis of high-risk NB. Tumors with *MYCN* amplification typically display increased proliferative capacity, resistance to differentiation signals, and altered metabolism. At the molecular level, *MYCN* drives the transcription of genes involved in cell cycle progression, ribosome biogenesis, and metabolic reprogramming. It also represses genes required for cellular differentiation and promotes genomic instability. *MYCN* amplification not only drives tumor proliferation and resistance to differentiation but also profoundly influences the tumor microenvironment (TME), contributing to immune evasion. Furthermore, *MYCN* cooperates with other oncogenic pathways, including Anaplastic lymphoma kinase (ALK) signaling and telomerase activation, to enhance tumor aggressiveness.

Cytokines are small secreted proteins, typically ranging from 8 to 12 kDa, that play essential roles in cell migration, adhesion and intercellular communication [4]. Their biological activity is mediated through binding to specific chemokine receptors. The cytokine-chemokine system comprises approximately fifty ligands and around twenty receptors, forming a complex network involved in numerous physiological processes, including development, immune homeostasis, inflammation, infection, and pathological conditions such as cancer [4]. In the context of tumorigenesis, chemokines can exert dual effects. On the one hand, they may directly influence tumor cells by promoting proliferation, invasiveness, and the maintenance of stem cell-like properties. On the other hand, they can act on stromal cells within the TME, thereby facilitating tumor invasion and metastasis [5]. Chemokines also play a pivotal role in modulating the immune response by regulating the recruitment and activation of immune cells at the tumor site [4]. However, their impact on cancer progression is context-dependent and may vary depending on the tumor stage, local immune composition, and microenvironmental cues [6]. Certain chemokine signaling pathways are under investigation due to their association with tumor regression. For example, receptors such as C-X-C motif chemokine receptor 1 (CXCR1) and CXCR3 have been linked to antitumor immune responses. Conversely, other chemokine receptors, including C-C chemokine receptor type 4 (CCR4) and CCR8, are being explored as potential targets for immunotherapy, as their expression is thought to contribute to immune suppression and tumor progression [7]. A comprehensive understanding of the cytokine and chemokine networks, as well as their interactions with the TME, is critical for the development of innovative and effective cancer therapies.

Recent studies have highlighted the role of chemokines in shaping the TME and promoting tumor progression. Among them, C-C motif chemokine ligand 2 (CCL2) has emerged as a pivotal immunomodulatory molecule. CCL2 regulates the recruitment of various immune cells, including monocytes, dendritic cells (DCs), invariant natural killer T (iNKT) cells, and regulatory T cells (Tregs), thereby influencing the balance between anti-tumor immunity and immunosuppression. Understanding the role of CCL2 may provide novel insights into the pathogenesis of NB and reveal new opportunities for therapeutic intervention, especially in the rising era of cellular immunotherapy.

## CCL2/CCR2 in cancer

**Fig. 1:**
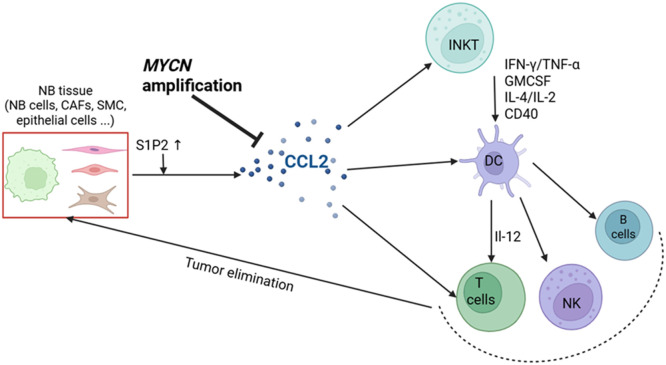
**Immunosuppressive Role of CCL2 in the Neuroblastoma Tumor Microenvironment**. CCL2 facilitates the recruitment of myeloid-derived cells (MDCs) and tumor-associated macrophages (TAMs) via the CCR2 receptor, as well as regulatory T cells (Tregs) and Th2-polarized CD4⁺ T cells through CCR4. Within the TME, CCL2 drives the differentiation of MDCs into immunosuppressive TAMs, which produce enzymes such as arginase-1 (ARG1) and inducible nitric oxide synthase (iNOS), both of which inhibit effector lymphocyte activity. TAMs further reinforce immunosuppression by secreting additional CCL2 and releasing soluble mediators that directly impair CD8⁺ T-cell cytotoxicity. In parallel, Tregs and Th2-associated cytokines skew CD8⁺ T-cell responses toward a dysfunctional, Th2-like phenotype, ultimately promoting tumor immune evasion and disease progression.

CCL2, also known as monocyte chemoattractant protein-1, is a chemokine expressed by a wide variety of tumors (breast, prostate, lung, glioblastoma, colorectal) which plays a central role in several tumor-associated physiological processes. CCL2 is particularly known for its ability to chemoattract monocytes, memory T lymphocytes, and DC to sites of inflammation or tumor growth, through interaction with its primary receptors, CCR2 and CCR4 [8]. The CCL2/CCR2 axis has been extensively studied due to its involvement in tumor progression, angiogenesis, and metastasis formation [9,10]. This signaling pathway is predominantly associated with immunosuppressive macrophages, particularly tumor-associated macrophages (TAMs), which express high levels of CCR2 [11]. Monocytes expressing CCR2 follow the CCL2 gradient to infiltrate the TME, where they differentiate into tumor-associated macrophages (TAM)s. Then they contribute to the establishment of an immunosuppressive TME (Figure 1). Furthermore, activation of the CCL2/CCR2 axis promotes the recruitment of Tregs, which are key players in suppressing anti-tumor immune responses within the tumor ecosystem [12]. Collectively, the CCL2/CCR2 axis constitutes a critical mechanism by which tumors shape their microenvironment to promote growth, invasion, and immune evasion, making it a promising target for the development of novel cancer immunotherapies.

## NB and immunity: the major role of TME

Like many other malignancies, NB has developed various immune evasion mechanisms allowing it to escape immune surveillance. One of the most well-documented strategies involves the downregulation of major histocompatibility complex class I (MHC-I) molecules, thereby limiting recognition by cytotoxic CD8+ T cells and impairing effective anti-tumor responses [13]. In addition, NB cells secrete a variety of immunomodulatory molecules, including cytokines and chemokines, which actively reshape the TME in favor of tumor growth and immune evasion. The main immune cells involved in this immunosuppressive TME are TAMs and Tregs. TAMs secrete numerous pro-tumoral factors, support angiogenesis, and suppress cytotoxic immune responses, making their presence a poor prognostic indicator in NB [14]. TAMs are key players in the inflammation and progression of NB. Depending on the cytokine milieu, macrophages can polarize into two major phenotypic states: M1 and M2. While M1 macrophages are generally pro-inflammatory and exert anti-tumoral functions, M2 macrophages—induced by cytokines such as IL-4, IL-10, and IL-13—exhibit pro-tumoral activity by promoting immune suppression, tissue remodeling, and tumor growth [14].

DCs are the primary antigen-presenting cells of the immune system and play a central role in the initiation of adaptive immune responses. They are crucial for T cell priming, leading to immune activation and tolerance. DCs are particularly important for the activation of CD8+ T cells and NK cells and are associated with favorable outcomes in several cancers, including NB [13,15]. However, in the TME of NB, various immunosuppressive factors such as IL-10, vascular endothelial growth factor, IL-6, and transforming growth factor-beta are secreted to inhibit DC maturation and function. This immunosuppressive environment impairs antigen presentation and limits effective T cell-mediated anti-tumor immunity. iNKTs are also of great interest in NB, they represent a small subset of lymphocytes, accounting for approximately 1% of peripheral lymphocytes. They share phenotypic and functional characteristics of both NK cells and conventional T lymphocytes, acting as a bridge between the innate and adaptive immune systems [16]. Importantly, iNKTs can recognize and kill tumor cells independently of MHC-I expression, which is particularly relevant in tumors such as NB that downregulate MHC-I. High levels of iNKTs have been associated with favorable prognosis in NB, correlating with spontaneous tumor regression and improved patient survival. Notably, they are capable of reprogramming or eliminating immunosuppressive cells such as TAMs, thereby restoring immune surveillance and enhancing tumor clearance [16].

The nature of the TME in NB appears to be closely associated with *MYCN* oncogene amplification. Tumors with *MYCN* overexpression are often characterized by a so-called "cold" TME, marked by low immune cell infiltration. In contrast, high-risk tumors lacking *MYCN* amplification may exhibit a "hot" TME, enriched in immune cells such as natural killer (NK) cells and CD8+ cytotoxic T lymphocytes, which are typically associated with a more active anti-tumor immune response [14,17]

## CCL2 and NB

**Fig. 2:**
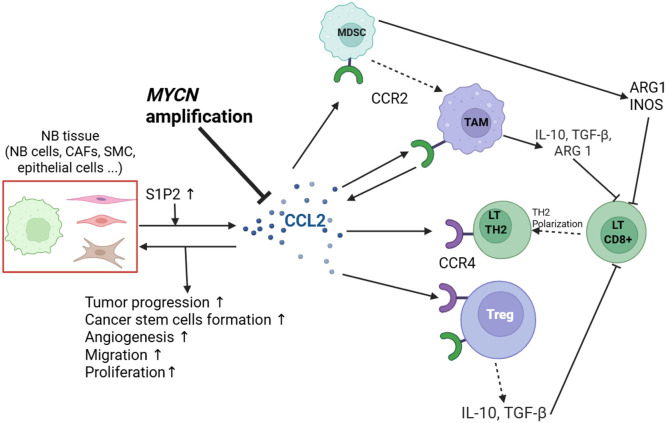
**Pro-Immunogenic Role of CCL2 in the Neuroblastoma Tumor Microenvironment**. Within the tumor microenvironment (TME) of neuroblastoma, tumor cells secrete the chemokine CCL2, potentially in response to sphingosine signaling mediated through the S1P2 receptor. CCL2 plays a central role in attracting immune cells—most notably invariant natural killer T (iNKT) cells—to the tumor site. Upon recruitment, iNKT cells release a variety of immunomodulatory cytokines, including IFN-γ, TNF-α, GM-CSF, IL-4, and IL-2, and initiate CD40-dependent signaling through interactions with dendritic cells (DCs). These signals promote the activation of DCs, which themselves migrate toward the tumor following the CCL2 gradient. Activated DCs subsequently produce IL-12, a key cytokine that enhances the cytotoxic activity of CD8⁺ T lymphocytes, natural killer (NK) cells, and supports B-cell responses, collectively fostering a robust anti-tumor immune response. Abbreviations: CAF, cancer-associated fibroblast; SMC, smooth muscle cells; S1P2, sphingosine-1-phosphate receptor 2.

Interestingly, in less aggressive NB, spontaneous tumor regression and cellular phenotype changes have occasionally been observed. In these cases, tumor cells may undergo transdifferentiation into fibroblast-like cells that strongly express CCL2 [18]. This phenomenon suggests a potential mechanism of tumor regression, whereby elevated CCL2 expression may enhance immune cell recruitment and promote tumor cell differentiation, ultimately leading to tumor elimination (Figure 2).

Given the multifaceted role of CCL2 in tumor biology, its implication in NB deserves particular attention. CCL2 expression is highly heterogeneous among NB tumors and appears to correlate with several key prognostic factors. Notably, *MYCN* amplification is inversely associated with CCL2 production, suggesting that high-risk, *MYCN*-amplified tumors tend to exhibit low CCL2 levels [19,20], by direct repression of CCL2 transcription [20,21]. CCL2 can support the survival of RA-treated neuroblastoma cells, highlighting a protumoral role, yet its induction does not fully prevent RA-mediated growth inhibition, reflecting a complex balance between pro-survival signaling and therapeutic differentiation effects [21]. *In vitro* studies reveal that knockdown of *MYCN* in amplified NB cell lines restores CCL2 production, thereby enhancing chemotaxis of various antitumor immune cells [20]. Correspondingly, NB tumors with *MYCN* amplification exhibit markedly lower CCL2 expression compared to non‑amplified tumors, a difference that correlates with significantly reduced immune cell infiltration [22]. Transcriptomic analyses further corroborate this inverse relationship; primary NB samples display a significant negative correlation between *MYCN* expression and CCL2 levels (Spearman r ≈ –0.5, P < 0.001) [22]. Functionally, impaired CCL2 secretion in *MYCN*‑amplified tumors disrupts recruitment of monocytes and DC (both myeloid and plasmacytoid subsets), which compromises subsequent T cell infiltration via secondary chemokines such as CCL19 and CCL22 [22]. Collectively, these findings demonstrate that *MYCN* amplification in NB not only drives tumor aggressiveness but also orchestrates immune evasion by downregulating CCL2, thereby dampening antitumor immune surveillance. This chemokine axis may represent a promising target for immunotherapeutic strategies aimed at restoring immune infiltration and enhancing anti‑tumor responses.

Therefore, the impact of CCL2 in NB remains context-dependent, and deciphering whether it exerts beneficial or detrimental effects is crucial for understanding its therapeutic potential.

In contrast, significantly elevated CCL2 secretion has been observed in tumors associated with opsoclonus-myoclonus syndrome, a paraneoplastic neurological disorder typically linked to low-risk NB [23]. These contrasting observations have led to conflicting interpretations of CCL2’s role in NB. On one hand, elevated CCL2 levels are found in low-risk tumors, suggesting a potentially beneficial or immunostimulatory role. On the other, CCL2 is also overexpressed in certain aggressive tumors lacking *MYCN* amplification, and numerous studies highlight its contribution to a pro-tumoral and immunosuppressive TME.

## CCL2 and iNKTs

iNKT and NK cells exert strong antitumor effects in NB by inhibiting TAMs and myeloid dendritic cells, as well as by directly eliminating cancer stem cells and targeting NB cells with downregulated MHC antigens. Several studies have demonstrated that CCL2 plays a key role in recruiting iNKT cells toward NB tumors [20].

Given the regulatory impact of MYCN on CCL2 expression, high-risk NB characterized by *MYCN* amplification suppresses iNKT recruitment. Indeed, the MYCN-high/CCL2-low phenotype accurately predicts the absence of iNKT cell infiltration [19]. This reduced recruitment of iNKT cells contributes to impaired antitumor immunity through an MHC-independent mechanism. While CCL2 promotes the beneficial recruitment of antitumor iNKT cells into neuroblastomas, its lower expression in MYCN-amplified tumors highlights a limitation, as insufficient CCL2 may prevent effective immune cell infiltration and antitumor immunity [19].

## CCL2 and DCs

DCs are indispensable for initiating adaptive immune responses through antigen presentation. Their migration and maturation in lymph nodes depend on CCR2, the receptor for CCL2. Upon CCL2 binding, CCR2 activates the NF-κB pathway, which drives the production of CCL19 and IL-12—two key mediators of immune activation and T cell recruitment. Moreover, NF-κB is essential for DC survival and functional maturation [22,24,25]. In NB, the presence of mature DCs correlates with favorable prognosis due to their capacity to activate cytotoxic T cells and coordinate immune responses [26].

CCL2 produced by MYCN-nonamplified neuroblastomas may support dendritic cell and monocyte infiltration, promoting T-cell recruitment and antitumor immunity, but its low expression in MYCN-amplified tumors limits immune cell infiltration and fosters a protumoral, immune-suppressive microenvironment, although this association remains controversial [22].

In other cancers such as breast and lung carcinoma, tumor-associated DCs can paradoxically secrete CCL2 themselves, promoting tumor progression rather than inhibition, by increasing cancer stem cell features, migration and invasion, and immunosuppressive TAM infiltration [24]. Thus, while CCL2 may facilitate DC recruitment, the resulting effect on tumor behavior depends heavily on the functional phenotype of the DCs and the surrounding TME. If this observation is also true in the case of NB, it reinforces the idea of CCL2′s contradictory effect on the tumor.

## CCL2 and T Lymphocytes

CCL2 drives CD4+ and CD8+ T-cell migration via chemotaxis, acting on a subset of CCR2-expressing T cells.

However, CCR2 regulation during T-cell activation remains controversial [12]. Some studies report its induction, while others describe downregulation, which may restrict responsiveness to CCL2 *in vivo*.

Although CCL2 efficiently recruits T cells *in vitro*, this effect is often diminished *in vivo* [27] due to competition with immunosuppressive myeloid cells, Treg interference, or receptor desensitization. Sustained high CCL2 levels can desensitize CCR2 signaling, reducing immune cell migration and tumor infiltration.

**Fig. 3:**
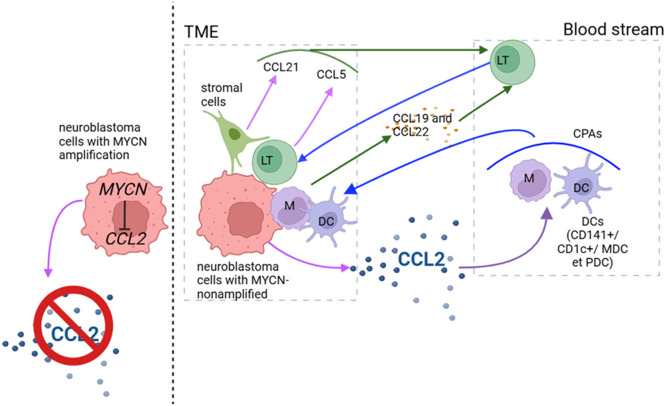
**Differential Regulation of CCL2 and Immune Cell Recruitment in MYCN-Amplified vs. Non-Amplified Neuroblastoma**. This figure illustrates how MYCN status in neuroblastoma cells shapes the chemokine landscape and thereby influences immune cell trafficking within the tumor microenvironment (TME). On the left, MYCN-amplified neuroblastoma cells suppress CCL2 expression, resulting in minimal recruitment of CCL2-dependent immune populations. In contrast, MYCN-non-amplified tumor cells in the TME secrete substantial amounts of CCL2, which promotes the recruitment of dendritic cells (DCs), monocytes (M), and other antigen-presenting cells (APCs) from the bloodstream into the tumor. Stromal cells within the TME additionally produce chemokines such as CCL21 and CCL5, further supporting the migration of lymphocytes (LT) toward the tumor. Circulating lymphocytes are also guided by CCL19 and CCL22 gradients toward the TME, contributing to immune cell infiltration. Various DC subsets (including CD141⁺, CD1c⁺, MDCs, and plasmacytoid DCs) are shown entering the tumor area, where CCL2 serves as a major chemoattractant. Overall, the figure highlights the contrasting chemokine environments driven by MYCN amplification status and underscores the central role of CCL2 in orchestrating immune cell recruitment into the neuroblastoma microenvironment.

The involvement of CCL2 in regulating T-cell responses in NB is still not well characterized. Several studies have reported a decreased T-cell infiltrate in highly aggressive tumors such as metastatic tumors [28]. A potential mechanism of action is expected to involve CCL2-mediated interactions between DC and T lymphocytes (Fig. 3) [22].

The CCR2/CCL2 axis is central to T lymphocytes recruitment, which improves the immune response, particularly in less aggressive NB. However, these effects could be controversial given that excess CCL2 could hinder their recruitment. Therefore, in cases of NB expressing high levels of CCL2 (low risk and high risk non-amplified), T lymphocytes risk partial inactivation by CCL2, and in NB expressing low levels of CCL2, there is a lack of recruitment and interaction with DCs. These observations tend to show that LTs may not be the major players in the immune response to NB.

## CCL2 and Tregs

CCL2 also contributes to immune evasion by modulating T cell polarization and promoting Treg recruitment [8]. High CCL2 expression has been associated with a shift toward T helper 2 (Th2) polarization, which is less effective in anti-tumor immunity [10,12]. Tregs, which express CCR4, are capable of binding CCL2, allowing them to home to CCL2-rich tumor sites and suppress local immune responses [8]. In gliomas and other tumor types, the CCL2/CCR4 axis has been identified as a key mechanism driving Treg accumulation in the TME.

In NB, while less well-characterized, similar mechanisms may be active, particularly in non-*MYCN*-amplified tumors.

Some studies suggest that CCL2 not only recruits Tregs but also enhances their functional interactions with TAMs, compounding the immunosuppressive effects within the tumor [12].

## CCL2 and myeloid derived cells (MDC)

Macrophages play a central role in the innate immune response by eliminating pathogens and abnormal cells and by orchestrating immune cell recruitment. In the tumor context, macrophages exhibit a remarkable plasticity, and their polarization into different functional states—most notably M1 and M2 phenotypes—greatly influences tumor progression [29,30]. M1 macrophages are pro-inflammatory and anti-tumoral, whereas M2 macrophages are involved in angiogenesis, immunosuppression, tumor growth, and metastatic spread. CCL2 is a well-established chemokine for the recruitment of monocytes into the TME, particularly those that give rise to pro-tumoral macrophages. Upon their arrival at the tumor site, monocytes differentiate into TAMs, which then support tumor proliferation and immune evasion. Numerous studies have demonstrated a positive correlation between CCL2 expression and TAM density in several malignancies, including breast and prostate cancer [8,31]. Moreover, TAMs themselves can secrete CCL2, establishing a feed-forward loop that sustains tumor-promoting inflammation. In glioblastoma, this feedback mechanism has been linked to the aryl hydrocarbon receptor expressed by TAMs, which enhances CCL2/CCR2 signaling and promotes macrophage accumulation [32]. Although direct evidence in NB is limited, TAMs are consistently observed in greater numbers in non-*MYCN*-amplified high-risk tumors, those also known to express higher levels of CCL2. This suggests a plausible role for CCL2 in TAM recruitment in NB as well.

One of the proposed mechanisms involves sphingosine-1-phosphate (S1P), a bioactive lipid that modulates immune signaling. Blockade of the S1P2 receptor has been shown to suppress CCL2 expression related to S1P in NB cells and decrease macrophage infiltration in xenografts [30], indicating that S1P signaling may regulate TAM infiltration via modulation of CCL2. CCL2 induced by S1P via S1P2 can positively recruit macrophages that may support antitumor immunity, but it may also promote a protumoral microenvironment, highlighting the dual role of CCL2 in neuroblastoma progression [30]

## Complexity of CCL2: Modulating factors

CCL2 has multiple actions he is well known for his actions in the immunity but can impact the tumor by other mean. For example activation of the signaling pathway with CCR2 contributes to tumor progression by inducing MMP2 and MMP9 expression within the tumor and its microenvironment, promoting extracellular matrix degradation and facilitating metastatic spread [10]. For instance, MMP12 can cleave CCL2, thereby converting it into a functional CCR2 antagonist [33].

The expression of CCL2 is also variable between the different stages of the tumor. Although tumor cells themselves can produce CCL2, its cellular origin varies with disease stage predominantly from NK cells and fibroblasts in newly diagnosed NB, and from endothelial, stem, and smooth muscle cells in refractory or relapsed cases [34].

The link between CCL2 and the different tumor classifications is one of the key points in understanding CCL2 and how to utilize it in NB. We’ve seen that CCL2 is overexpressed in low-risk and high-risk NB not amplified for MYCn. This observation raises questions about the treatment of high-risk NB with amplified MYCn. It is therefore logical to assume that if treatments using CCL2 are introduced into clinical practice, the patient's MYCn status will need to be rigorously analyzed to avoid administering an ineffective treatment.

On the one hand, it would be interesting to study whether the addition of CCL2 in these tumors would restore a TME that is better able to defend itself against the tumor. To do this, devices such as oncolytic viruses or fourth-generation chimeric Antigen Receptor T (CAR-T cells) modified to express the chemokine could increase CCL2 levels around the tumor and promote immune defense.

On the other hand, certain treatments such as retinoic acid or anti-GD2 antibodies are known to increase CCL2 secretion, which may be linked to resistance phenomena and the recruitment of immunosuppressive immune cells. In this case, treatment in combination with antibodies or CCL2 inhibitors could increase the efficacy and longevity of the treatment.

Specific study of these problematic NB could provide definitive answers as to whether this chemokine is beneficial or harmful.

## CCL2 as a Therapeutic target and tool

The role of CCL2 in NB has also been investigated in the context of immunotherapy. The anti-GD2 antibody ch14.18, widely used in NB treatment, induces antibody-dependent cellular cytotoxicity, which in turn stimulates the release of various cytokines and chemokines, including CCL2 [35].

One of the new therapeutic strategies in neuroblastoma is the use of retinoic acid [36] as a differentiation therapy to reduce MYCN expression in tumors [21]. In this context, the gene encoding CCL2 has been identified as a key target. Retinoic acid treatment increases CCL2 secretion in neuroblastoma cell lines by modulating specific signaling pathways, which may contribute to the emergence of cells resistant to retinoic acid. These findings indicate that analysis of MYCN and CCL2 levels alone cannot reliably predict tumor cell fate following treatment [21]. CAR-T cell therapy represents a promising approach for solid tumors, including NB. Recent studies have explored the genetic modification of CAR-T cells to express CCR2, enhancing their chemotactic response to CCL2 gradients [37]. In preclinical models of metastatic non-small cell lung cancer, CCR2-overexpressing CAR-T cells demonstrated improved tumor infiltration and efficacy, including in brain metastases. These findings raise the possibility of applying similar strategies in NB, particularly in tumors with high endogenous CCL2 levels.

## Therapeutic target against CCL2

Given its central role in immune modulation and tumor progression, CCL2 is an attractive therapeutic target. Targeting CCL2 could be an efficient way to reduce the impact of the TAM and the pro inflammatory monocytes on the TME of NB [38]. Therapeutic approaches targeting TAMs have shown good results on other tumors [39,40]. One study demonstrated that inhibition of carbonic anhydrase IX (CA IX), a hypoxia-associated enzyme, can reduce CCL2 expression in NB [41]. This downregulation was associated with decreased tumor progression and altered TME signaling. However, the study did not evaluate changes in immune cell populations following treatment, leaving open questions about the broader immunological consequences of CA IX inhibition.

A prime example of the duality of CCL2 action and treatment effectiveness is the use of anti-CCL2 antibodies. These antibodies have no direct effect on cytotoxicity or cell proliferation; however, they significantly reduce the chemotaxis of immune cells. This demonstrates that CCL2 primarily acts via CCR2 on the surface of immune cells [42]. Consequently, the antibody has a negative effect by reducing the migration of T cells and NK cells, but also a positive effect by limiting the recruitment of monocytes and their differentiation into tumor-associated macrophages (TAMs), which contribute to angiogenesis, metastasis, and immunosuppression.

Monotherapy with anti-CCL2 antibodies clearly has limitations. However, combination therapy with chemotherapy and anti-CCL2 antibodies could reduce tumor microenvironment–induced resistance and improve efficacy. In vivo, the combination of etoposide and caluximab has been shown to significantly increase overall survival in mice compared to either monotherapy [42].

Targeting CCL2 directly, or the signaling axis (e.g., CCR2/CCR4), remains a promising strategy. Several experimental therapies, including monoclonal antibodies and small-molecule inhibitors, are being explored in preclinical models on other types of cancer.

At present, CCL2 remains a promising preclinical target in neuroblastoma (NB), particularly in the context of minimal residual disease and metastasis in CCL2-high, MYCN-non-amplified tumors. However, this has not yet translated into clinical trials evaluating CCL2 blockade in NB. To date, only preclinical studies of CCL2 inhibition have been conducted in NB, with no clinical trials currently available. In contrast, several clinical studies targeting the CCL2/CCR2 axis have been performed in other cancers and inflammatory diseases, providing important insights for potential therapeutic strategies. Carlumab, a monoclonal antibody directed against CCL2, demonstrated effective target engagement in tumor settings [43]. However, it failed to show clinical benefit compared to placebo in idiopathic pulmonary fibrosis, and some study arms were discontinued due to worsening patient conditions [44]. Similarly, ABN912, another anti-CCL2 antibody, proved ineffective in patients with rheumatoid arthritis [45]. These findings suggest that direct CCL2 inhibition may not be sufficient to achieve meaningful clinical outcomes. In contrast, CCX872, a selective CCR2 antagonist that blocks the CCL2/CCR2 axis, has shown more encouraging results. In pancreatic cancer, particularly in combination with the chemotherapy regimen FOLFIRINOX, CCX872 was associated with improved survival outcomes and a reduction in peripheral blood mononuclear cell (PBMC) counts [46]. This highlights the potential benefit of targeting CCR2 rather than CCL2 directly, especially in combination with standard therapies. Overall, clinical outcomes with direct CCL2 inhibition have been disappointing, with no significant therapeutic benefit observed. One possible explanation is that CCL2 blockade may impair the recruitment of key immune effector cells, such as T lymphocytes, natural killer cells, and macrophages, thereby leading to unintended deleterious effects. By contrast, CCR2 inhibition—particularly when combined with chemotherapy—may enhance therapeutic efficacy while avoiding some of these limitations. Although CCL2 blockade has not yielded satisfactory results in adult diseases, these findings may still inform NB research. Future strategies could focus on combination therapies with established chemotherapeutic agents or on more selective targeting of CCR2. Importantly, CCL2 can also signal through alternative receptors such as CCR4, particularly in regulatory T cells (Tregs). This suggests that even when the CCL2/CCR2 axis is inhibited, residual CCL2 activity may persist through CCR4, potentially limiting treatment efficacy. In this context, combination approaches integrating CCL2/CCR2 axis modulation with chemotherapy or immunotherapy may be required to overcome resistance mechanisms and improve outcomes.

## Conclusion

CCL2 plays a complex and context-dependent role in NB, influencing tumor progression largely through modulation of the TME. By promoting the recruitment of immunosuppressive cells such as TAMs and Tregs, while simultaneously affecting the activity of DC, T lymphocytes, and iNKT cells, CCL2 contributes to immune modulation of TME surrounding NB. Moreover, *MYCN* amplification in NB emerges not only as a key driver of tumor aggressiveness but also as a critical modulator of the TME immunity through the suppression of CCL2 expression. This downregulation of CCL2 impairs the recruitment of immune effector cells, facilitating immune escape and contributing to disease progression. Targeting this chemokine axis could therefore represent a novel and promising immunotherapeutic approach, with the potential to enhance immune cell infiltration and improve antitumor immunity in *MYCN*-amplified NB.

## CRediT authorship contribution statement

**Léo Jannot:** Writing – review & editing, Writing – original draft, Validation, Supervision, Methodology, Formal analysis, Conceptualization. **Alexia Gazeu:** Writing – review & editing, Validation, Methodology. **Nathalie Bendriss-Vermare:** Writing – review & editing, Writing – original draft, Validation, Supervision, Methodology. **Cecile Pochon:** Writing – review & editing, Writing – original draft, Validation. **Hervé Sartelet:** Writing – review & editing, Writing – original draft, Validation, Supervision.

## Declaration of competing interest

The authors declare that they have no known competing financial interests or personal relationships that could have appeared to influence the work reported in this paper.
